# Well-Balanced Lunch Reduces Risk of Lifestyle-Related Diseases in Middle-Aged Japanese Working Men

**DOI:** 10.3390/nu13124528

**Published:** 2021-12-17

**Authors:** Mari Mori

**Affiliations:** Department of Health Management, School of Health Studies, Tokai University, 4-1-1 Kitakaname, Hiratsuka 259-1193, Kanagawa, Japan; m_mori@tsc.u-tokai.ac.jp; Tel.: +81-463-63-4815

**Keywords:** nutritional biomarkers of 24-h urine, soy isoflavones, DHA, optimal salt, sodium/potassium ratio, atherogenic index, lunch intervention

## Abstract

Based on the results of a previous WHO-CARDIAC study, this study was designed to test the effect of the daily consumption of a diet rich in potassium with optimal salt content, rich in fish meat and soy isoflavones, corresponding to the ingredients of a traditional Japanese diet. The test meals were a Balanced Lunch (BL) with chicken as the main dish and a Fortified Lunch (FL) with fish and soy as the main dish, which appeared the same. A double-blind, randomized controlled trial was conducted in 64 relatively obese men (47.2 ± 5.5 years old) who consumed the lunch at their work site for 4 weeks. All participants underwent fasting blood sampling, 24-h urine collection, as well as measurements of height, weight, and blood pressure before and after the intervention. Body mass index, blood pressure, and HbA1c were significantly improved and a 3-g reduction of salt intake was proven by 24-h urine collection in both groups. Moreover, HDL cholesterol and the Atherogenic Index (AI) were significantly improved in the FL group. In conclusion, the risks of lifestyle-related diseases in working men were reduced by one meal per day intervention of optimally-salted traditional Japanese diets containing soy and fish nutrients with high vegetable ingredients.

## 1. Introduction

Lifestyle-Related Diseases (LRD) developing by gene-environmental interaction, are epidemiologically prevented by optimizing nutritional intake, based on the data obtained by our CARDIAC study [1,2,3,4]. In particular, soybeans which have been traditionally eaten in Japan, are expected to prevent LRD such as stroke and coronary heart diseases [5]. In addition, seafood, which is obtained from the surrounding sea and is eaten regularly, has also been shown to prevent LRD as well [1,6]. In this study, a nutritional intervention trial was conducted to confirm whether daily intake of soy isoflavones from soybeans and Docosahexaenoic Acid (DHA) from fish as food could have a preventive effect on LRD in working-age men who are relatively at high risk of these diseases. As a method of nutritional intervention, a special lunch menu (called the “One Meal a Day” project in this study) enriched with soybean isoflavones and fish DHA based on the results of the CARDIAC study, was designed with optimal salt, fat ratio, and energy content, and the participants were provided a daily lunch after their informed consent. Cardiovascular risk factors such as BMI, blood pressure, blood lipid-related indices, and Atherogenic Index (AI) were targeted as study outcomes. Sodium excretion, sodium to potassium ratio, and soy isoflavones were estimated as nutritional biomarkers of a 24-h urine sample to confirm the effect of dietary intake.

## 2. Materials and Methods

### 2.1. Participants and Methods

The subjects of this study were 64 males (47.2 ± 5.5 years old) over 40 years old, working in an IT company (Table 1), who were relatively at high risk of LRD. They were randomly assigned to two groups: A balanced diet (BL: *n* = 32) and a fortified diet (FL: *n* = 32), in which BL was fortified with fish and soy nutrients. All these meals were served at lunch. Lunch was consumed for 4 weeks, and the effects of lunch consumption were assessed by 24-h urine collection and physical examination before and after the intervention.

### 2.2. Study Design

This study was conducted as a double-blind randomized controlled trial. Participants were recruited from people relatively at high risk of LRD, and the principal investigator conducted a briefing session on the purpose and content of the study. Participants who were not allergic to any of the foods in the lunch were invited to consume the lunch provided every day for 4 weeks. The exclusion criteria were those who had been diagnosed with LRD (e.g., hypertension or diabetes), and those who were taking supplements or medications.

Participants were randomized into two groups by a person not associated with the study, based on the pre-intervention data. They were asked to continue to live a normal life during the intervention period and to limit their intake of binge drinking, soy products, and seafood other than the test meal.

This study was conducted with the approval of the Ethics Committee (no. 04-0201) of the Institute for Health Restoration, Inc.

### 2.3. Test Meals

Two types of test diets were prepared: A Balanced Lunch (BL) with good nutritional balance and a Fortified Lunch (FL), which was a BL fortified with soy and fish nutrition. The standard for lunch was set at 750 kcal or less energy corresponding to 1/3 of the daily dietary intake standard for the subject. Salt content for the lunch was less than 2.5 g, and the fat to energy ratio was less than 30%. We then prepared 10 different lunches that met these standards (Table 2).

The main meal and side dishes of BL and FL were similar, but the main dish of BL consisted of chicken, and the main dish of FL was fish and soybean and was contained more than 700 mg of polyunsaturated fatty acids (DHA) and 40 mg of isoflavones. The appearance of the two types of test meals was the same. Therefore, to avoid any mistakes regarding the meal, the research collaborators handed the lunches directly to the participants according to their IDs and collected the empty containers to record the remaining food after lunch. The participants were also instructed to keep a dietary diary during the intervention period for recording their leftover lunch, as well as soybean and seafood intake outside of lunch.

### 2.4. Medical Examination

Before and after the 4-week lunch intervention, health screenings were conducted at the participants’ working site. On the day before the medical checkup, dinner was finished by 9:00 p.m., and consumption of food and beverages other than water after that time was prohibited. On the morning of the health checkup, the participants gathered on empty stomachs, and after height and weight checks and 15 min of sitting, blood pressure was measured by OMRON HEM-907. After that, blood was drawn. After the medical checkup, we distributed 24-h urine collection containers and explained how to collect urine. After being given the instructions, the patients started collecting urine in 24-h aliquot cups (manufactured by Izumi Co., Ltd., Hiroshima, Japan) used for the WHO-CARDIAC study.

Blood samples were analyzed for Total Cholesterol, HDL-cholesterol, Triglyceride, Glucose, HbA1c, Insulin, and AI was calculated as the non-HDL/HDL ratio. The 24-h urine sample was analyzed for sodium, potassium, creatinine, magnesium, urea nitrogen, isoflavones, and taurine, and salt and protein intakes (g) were calculated from sodium and urea nitrogen excretion in 24-h urine.

### 2.5. Statistical Analysis

The measurements obtained were presented as mean ± standard deviation. The student’s *t*-test was used to compare the pre- and post-intervention values for each test diet group, and for inter-group comparisons of the evaluation items, the amount of change before and after the intervention was calculated and tested using the student’s *t*-test. The significance level for both tests was set at 5%, and IBM SPSS Statistics Ver. 24 was used for statistical processing.

In addition, those who were unable to participate in the 24-h urine collection and medical checkups before and after the intervention, and those who consumed less than 75% of the lunch were excluded from the analysis. A total of 64 subjects (47.2 ± 5.5 years old), who gave written consent, were included in this study.

## 3. Results

### 3.1. Characteristics of the Participants

**Figure 1:**
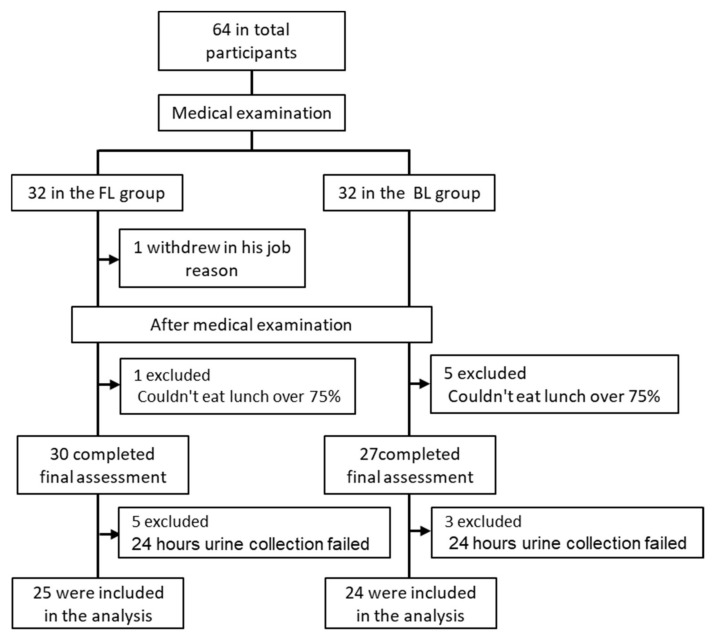
Enrollment of the participants and completion of the study. FL: Fortified Lunch group and BL: Balanced Lunch group.

Of the 64 participants who gave consent, 49 were included in the analysis, excluding 1 participant who was unable to attend the post-intervention checkup due to a business trip or other schedule, 6 participants whose lunch intake rate was less than 75% due to work-related reasons, and 8 participants who were unable to successfully collect urine for 24 h before and after the intervention (Figure 1).

### 3.2. Changes in BMI, Blood Pressure, and Blood Sampling Test

**Figure 2:**
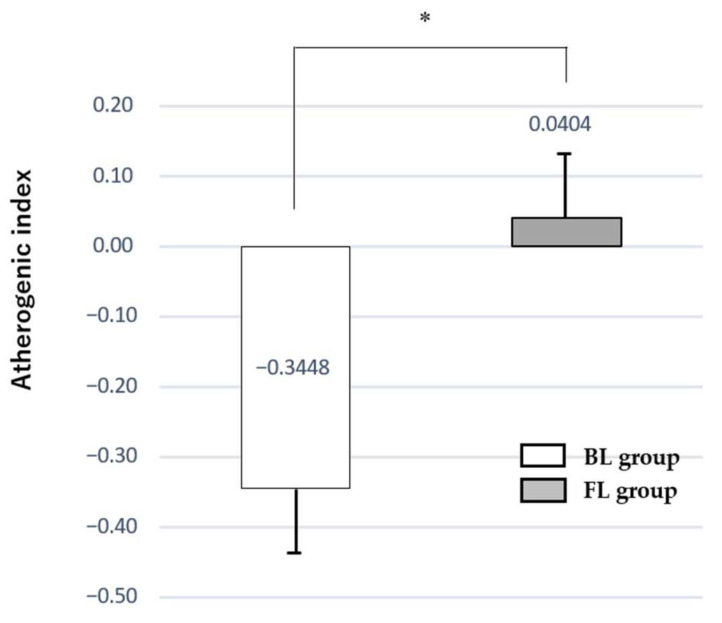
Comparison of Changes from the baseline in Atherogenic index (AI) in the FL and BL after 4-week intervention. AI: Non-HDL-cholesterol/HDL-cholesterol, there was significant group difference between the FL and BL group in changes in AI. *: *p* = 0.016.

The results before and after the intervention are shown in Table 3. Baseline data showed no significant difference between the FL and BL groups. After the intervention, body weight was significantly (*p* < 0.001) lower and BMI (kg/m^2^) significantly (*p* < 0.001) decreased in both groups. In terms of blood sampling, HbA1c showed a significant (*p* = 0.03 in FL), (*p* < 0.001 in BL) improvement in both groups. In the BL group, Diastolic Blood Pressure (DBP) was significantly (*p* = 0.002) improved than baseline levels, while in the FL groups, DBP (*p* = 0.02), HDL cholesterol (*p* = 0.01), and AI (*p* = 0.003) were significantly improved. Furthermore, there was a significant inter-group difference (*p* < 0.05) in the change from the baseline levels in AI between the FL and BL groups, as the FL group improved significantly after the intervention (Figure 2).

### 3.3. Changes in Nutrition Biomarker of 24-h Urine Sampling

The results of 24-h urine collection showed significant improvement in salt intake (g) in both the FL group (*p* < 0.01) and BL group (*p* < 0.03). In the FL group, the sodium to potassium ratio improved significantly (*p* = 0.01), and isoflavones showed a significant (*p* < 0.001) increase. The excretion of magnesium (*p* < 0.01) and protein (*p* < 0.04) was significantly lower in the BL group than baseline levels (Table 3).

### 3.4. Impressions of Participation in the Study and Evaluation of Optimal Salt Diet

Questionnaires were administered to all study participants after the intervention study. There were two main questions: (A) How did you feel about participating in the study, and (B) would you buy the lunches used in the study if they were available for sale?

**Figure 3:**
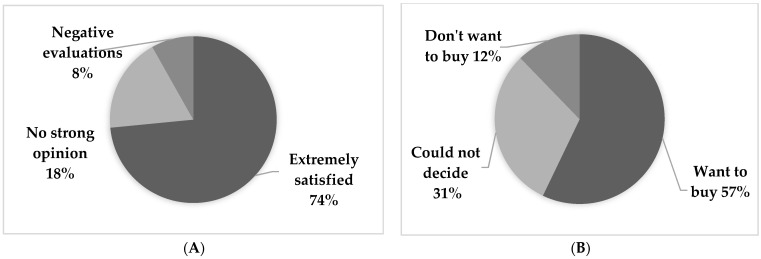
Impressions of participation in this study in response to questions: (**A**) How did you feel about participating in the study; and (**B**) would you buy the lunches used in the study if they were available for sale?

The results are shown in Figure 3. In response to (A), 74% of the participants said they were happy to have participated and commented the following: “I became more health conscious by eating nutritionally balanced lunches with the optimal amount of salt for 4-weeks”, “I learned how to eat optimally”, and “I got used to eating less salt”. The consumption of nutritionally balanced lunches was linked to nutrition education for the participants. On the other hand, 8% of the respondents gave negative evaluations, such as the meals given to them every day were boring.

Regarding the answer to (B), 57% of the respondents indicated that they would like to purchase the lunches, while 31% could not decide. The breakdown showed that many respondents would consider the lunches if their own health checkup results had improved, and 88% of respondents confirmed that they had a favorable impression of the lunches, while the remaining 12% of respondents did not want to use the lunches.

## 4. Discussion

The study showed that consuming even just one nutritionally balanced BL a day improved their BMI, blood pressure, and HbA1c, and on top of that, FL with foods that have been consumed traditionally since ancient times in Japan, such as fish and soybeans, improved lipoprotein profiles [7,8,9]. This is the first validation that shows that subjects at high risk of LRD can efficiently reduce their risk of cardiovascular diseases, such as stroke and coronary heart diseases, and LRD, even in a short period of 4 weeks, by consuming just once a day an optimized lunch instead of the entire daily diet [7,8,10].

### 4.1. Optimized Lunch and Its Effects

The lunch used in this study had an optimized energy level 750 kcal or less at one-third of the daily energy intake of working men with office jobs and contained 2.5 g or less salt. In addition, because of the large amount of side dishes, about 8 g of dietary fiber, the target amount for one meal, was contained. Moreover, the main dish in the FL group was not derived from meat, but from fish, which was effective in improving, lipid profiles [6,7,8] and HDL functionality [9], and FL contained isoflavones, soybean polyphenols [5], which worked to improve the blood lipid profile [8,9], and even significantly improved AI. These results support that the lunch containing the appropriate amount of energy, good fats, and dietary fibers optimized for the subjects improved their lipid profile, attenuating overall obesity to lower blood pressure and cardiovascular and diabetic risks [10].

However, there are reports that n3 fatty acid intake improved blood glucose, insulin, and HOMA-IR levels and AI in diabetic patients [11], but in the present study, there was no improvement in blood glucose, insulin, or HOMA-IR levels in the FL group, even though HbA1c improved in both groups. Despite the improvement in HbA1c, some subjects had higher blood glucose and insulin levels, which may be due to the fact that blood glucose and insulin are affected by the previous day’s meal.

### 4.2. Changes in Nutritional Biomarkers by 24-h Urine Collection

By having the participants consume a diet with salt content adjusted to 2.5 g or less as lunch for 4 weeks, salt intake, estimated by 24-h urine collection, was reduced from 14.1 to 11.2 g in the FL group and from 14.1 to 11.0 g in the BL group. Therefore, a reduction of 3 g was achieved in both groups. A previous WHO-CARDIAC study showed that daily salt intake less than 7 g was associated with nearly almost zero stroke mortality [2,3,12]. For the participants in the present study to succeed in reducing salt down to 7 g per day, they should reduce salt by 7 g from 14 g before intervention. By consuming the optimal amount of salt in a lunch for 4 weeks, they could actually reduce their salt intake by 3 g per day, from 14 down to 11 g, which might correspond to a nearly 40% reduction in stroke mortality, if such an optimum lunch could be continued throughout a person’s life.

In a questionnaire survey of the participants after the study, many commented that they were able to get used to the light taste of the food. This data suggests that if they can continue eating the optimal amount of salt for a longer period of time, they may be able to feel that the food with the optimal amount of salt tastes good and may achieve a salt intake of 7 g per day, which would reduce the mortality rate of stroke due to salt intake to nearly zero. The significant increase in urinary isoflavones in the FL group confirmed that they were getting enough isoflavones fortified in the main dish for lunch. The reason why the amount of potassium and magnesium did not increase despite the inclusion of sufficient side dishes in the lunch may be that these minerals are water soluble and may have been lost in the processing stage, particularly because frozen and processed vegetables in the ingredients were used to save the cost of commercial lunch preparation.

In Europe and the United States, where there are many obese people, the Dietary Approach to Stop Hypertension (DASH) diet, which includes fruits, vegetables, and low-fat dairy products, has been advocated, and the DASH Collaborating Research Groups conducted a study to prove the antihypertensive effect of eating the DASH diet every day [13]. In addition to the DASH diet, it was proven that reducing salt in a diet enhanced the antihypertensive effect [14]. The DASH diet itself was originally developed to prevent high blood pressure, but it was also effective against obesity due to its high dietary fiber content. In a previous study conducted in a Japanese version of the DASH diet, consuming three meals a day from a menu based on the DASH diet not only reduced salt intake, but also significantly improved serum lipids, such as total cholesterol and LDL cholesterol [15]. However, changing all three meals a day requires large changes in diet, making it difficult to continue. Therefore, a study was conducted in which the DASH diet was incorporated into one or two meals a day. Although a trend towards a reduced risk of LRD was observed, no significant difference was found in these trials [16]. The lunch provided in the present study was a menu that almost met the nutritional value of one serving of the DASH diet standard (Table 2). The improvement in AI observed after only 4 weeks of eating the FL diet, enriched with fish and soybean nutrients, is supposed to be due to the combined effect on HDL elevation by soy isoflavones and the sufficient supply of n3 fatty acid. The further studies are needed about the mechanism of this possible combined nutritional effect on AI and the functionality of HDL [9].

### 4.3. Strengths and Limitations

The strengths of this study are, firstly, we were able to conduct a double-blind lunch intervention in the workplace for working males at a relatively high risk for LRD without lifestyle restrictions, secondly a 24-h urine collection survey was used to assess the nutrition of the subjects, thirdly we were able to conduct medical examinations in the workplace, and fourthly the research staff was able to distribute lunch boxes without error and check the leftover food by collecting the containers directly after lunch every day.

Limitations were that we only restricted overeating and drinking during intervention period and did not restrict breakfast and dinner. Secondly, a few participants were unable to consume lunch because of their work, and thirdly some participants failed to collect 24-h urine.

The lunch was provided for 4 weeks and the effects of daily consumption of fish meat and soy isoflavones as the main dish were examined in this study. Both FL and BL groups had eaten the optimized lunch with adjusted energy, salt, and fat levels and resulted in the risk reduction of LRD. Participants in the FL group showed that the lipid profile improved significantly to reduce the risk of atherosclerosis. The reduction in the risk of LRD observed in this study could be attributed not only to eating a balanced lunch, but also to the effect of nutrition education by eating an optimized lunch. However, further research is needed to evaluate the effect of dietary education in this intervention study.

## 5. Conclusions

The daily intake of a nutritionally balanced lunch containing soy and fish nutrients was proven to reduce the risk factors of LRD. A traditional Japanese diet, rich in soybeans and seafood are associated with lower cardiovascular risks epidemiologically if salt intake is optimized [4,8,10]. In this study, an optimal, low-salt, well-balanced soy and fish diet by just one serving per day was enough to provide a variety of health benefits such as reducing obesity and blood pressure, as well as improving lipid profile and AI. To reduce the risk of LRD, especially in urban areas, the food industry should provide such healthy meals for consumers to make them easily accessible. This study indicates that consuming traditional Japanese food once a day with optimal salt content, which contains soy and fish nutrients rich with vegetables, can contribute to reducing LRD risk, improving health, and promoting longevity.

## Figures and Tables

**Table 1:** Baseline characteristics of the study participants.

	Test Group (*n* = 32)	Control Group (*n* = 32)
Age (years old)	47.2 ± 5.6	47.2 ± 5.5
Height (cm)	171.0 ± 5.6	169.6 ± 5.7
Weight (kg)	73.5 ± 9.9	72.1 ± 10.1
BMI (kg/m^2^)	25.1 ± 2.7	25.1 ± 3.2
SBP (mm Hg)	126.5 ± 15.6	125.5 ± 11.3
DBP (mm Hg)	80.3 ± 11.3	79.5 ± 9.1

Data are means ± SD, there were no significant differences between the FL vs. BL group.

**Table 2:** Menu and nutritional value used in this study.

Menu		Nutritional Value			Menu		Nutritional Value		
			FL	BL				FL	BL
Hamburger vegitable sauce 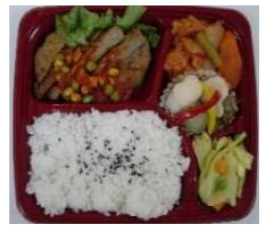	Rice Hamburger vegitable sauce Boiled vegetable (Chinese cab-bage/cauliflower/carrot, etc.) Vegetable marinade Cabbage onion curry sauce	Energy (kcal)	708	713	Ground Meat Cutlet 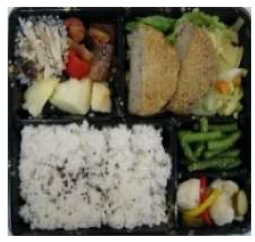	Rice Ground Meat Cut-let Cabbage onion curry saute Boiled eggplant Mushroom saute Kofuki potato Mixed with green beans Vegetable marinade	Energy (kcal)	717	723
		Protein (g)	24	20			Protein (g)	22	17
		Lipid (g)	24	21			Lipid (g)	25	20
		Carbohydrate (g)	80	109			Carbohydrate (g)	100	118
		K (mg)	760	713			K (mg)	907	790
		Dietary fiber (g)	11.1	7.4			Dietary fiber (g)	11.6	8.0
		NaCl (g)	0.9	1.2			NaCl (g)	2.0	2.2
		DHA (mg)	980	-			DHA (mg)	882	-
		Isoflavone (mg)	40	-			Isoflavone (mg)	40	-
Deep-fried lotus root 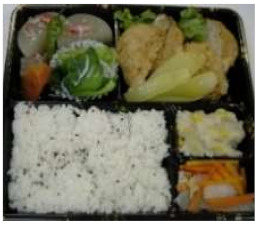	Rice Deep-fried lotus root Curry onion Wax gourd Deep-fried meat Boiled food (car-rot, konjac Bok choy with chirimen Chinese cabbage vinegar	Energy (kcal)	718	720	Hamburger tomato sauce 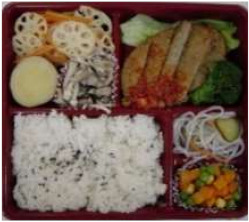	Rice Hamburger tomato sauce Broccoli Lotus root Kinpira Mushroom saute Sweet potato simmered inlemon Konjac vinegared food Vegetable con-somme boiled	Energy (kcal)	740	744
		Protein (g)	24	21			Protein (g)	25	20
		Lipid (g)	21	14			Lipid (g)	24	22
		Carbohydrate (g)	105	124			Carbohydrate (g)	104	117
		K (mg)	970	942			K (mg)	938	842
		Dietary fiber (g)	10.8	7.8			Dietary fiber (g)	12.3	8.6
		NaCl (g)	1.8	2.3			NaCl (g)	2.5	2.8
		DHA (mg)	827	-			DHA (mg)	980	-
		Isoflavone (mg)	40	-			Isoflavone (mg)	40	-
Tsukune 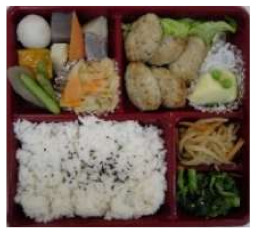	Rice Tsukune Potato and Shirataki noodles Boiled food (potato/pumpkin/carrot/konjac/bamboo shoot/burdock/bean) Jellyfish vinegared Kiriboshi daikon Boiled spinach	Energy (kcal)	727	735	Deep-fried lotus root 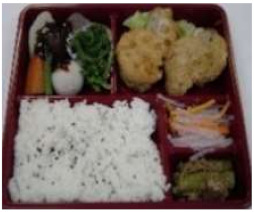	Rice Deep-fried lotus root Boiled vegitable Dengaku (Radish/Konjac/Carrot/Small potato/Ingen/Shiitake mushroom) Agar vinegared food Kiriboshi daikon Boiled butterbur with bonito	Energy (kcal)	720	723
		Protein (g)	26	22			Protein (g)	23	20
		Lipid (g)	23	17			Lipid (g)	20	14
		Carbohydrate (g)	101	121			Carbohydrate (g)	110	128
		K (mg)	1258	1136			K (mg)	867	839
		Dietary fiber (g)	8.9	10.0			Dietary fiber (g)	11.0	8.0
		NaCl (g)	2.8	3.0			NaCl (g)	2.2	2.6
		DHA (mg)	1157	-			DHA (mg)	827	-
		Isoflavone (mg)	40	-			Isoflavone (mg)	40	-
Pepper stuffed with meat 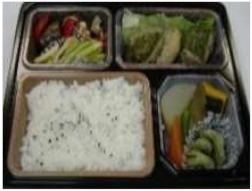	Rice Pepper stuffed with meat Boiled eggplant Grilled white onion Boiled food (radish/pumpkin/burdock/turtle) Kiriboshi daikon Boiled broad bean waste	Energy (kcal)	651	649	Tsukune sweet and sour sauce 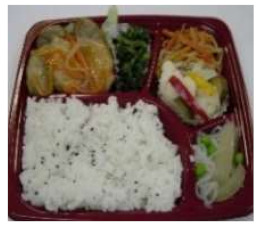	Rice Tsukune sweet and sour sauce Kiriboshi daikon Kinpira Vegetable marinade Onion Shirataki Kiriboshi daikon	Energy (kcal)	725	718
		Protein (g)	23	22			Protein (g)	24	21
		Lipid (g)	19	11			Lipid (g)	24	16
		Carbohydrate (g)	94	107			Carbohydrate (g)	102	122
		K (mg)	791	841			K (mg)	1448	1326
		Dietary fiber (g)	10.5	6.8			Dietary fiber (g)	14.4	11.5
		NaCl (g)	2.2	2.1			NaCl (g)	2.1	2.3
		DHA (mg)	892	-			DHA (mg)	1157	-
		Isoflavone (mg)	40	-			Isoflavone (mg)	40	-
Chicken balls with tomato 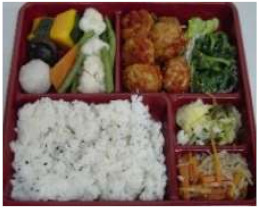	Rice Chicken balls with tomato Boiled rape blossoms Boiled food (Pumpkin/Shiitake mushroom/Small potato/Carrot) Cauliflower and green beans mixed with sesame seeds Chinese cab-bage trefoil vinegared Konjac Kinpira	Energy (kcal)	711	718	Pepper stuffed with meat 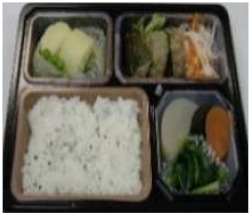	Rice Pepper stuffed with meat Red and white Boiled potatoes and onions Boiled food (radish, carrot, shiitake mushroom) Boiled Rape blossoms	Energy (kcal)	684	678
		Protein (g)	25	21			Protein (g)	22	22
		Lipid (g)	20	18			Lipid (g)	19	14
		Carbohydrate (g)	106	119			Carbohydrate (g)	107	120
		K (mg)	958	940			K (mg)	780	831
		Dietary fiber (g)	12.1	8.6			Dietary fiber (g)	11.1	7.4
		NaCl (g)	2.1	2.4			NaCl (g)	2.6	2.5
		DHA (mg)	700	-			DHA (mg)	892	-
		Isoflavone (mg)	40	-			Isoflavone (mg)	40	-

Energy < 750 kcal, salt < 2.5 g in both BL and FL. Fat energy ratio: BL < 25%, FL < 30%. Only FL: DHA > 700 mg, isoflavone > 40 mg.

**Table 3:** Changes from the baseline data after 4-week intakes of Fortified Lunch (FL) and Balanced Lunch (BL).

	Fortified Lunch Group (n25)			Balanced Lunch Group (n24)		
	Baseline	4 Weeks	*p*-Value	Baseline	4 Weeks	*p*-Value
Physical examination and blood pressure						
Age (years old)	47.1 ± 6.1			46.0 ± 4.3		
Height (cm)	170.3 ± 5.5			170.4 ± 5.9		
Weight (kg)	73.3 ± 8.8	72.2 ± 8.7	<0.001	72.4 ± 10.7	71.5 ± 10.7	<0.001
BMI (kg/m^2^)	25.2 ± 2.6	24.9 ± 2.5	<0.001	25.0 ± 3.5	24.7 ± 3.5	<0.001
SBP (mm Hg)	125.6 ± 12.5	125.6 ± 14.4	0.99	126.3 ± 12.3	122.4 ± 14.4	0.06
DBP (mm Hg)	79.6 ± 10.8	76.1 ± 12.0	0.02	80.5 ± 9.9	77.0 ± 11.8	0.002
Blood sampling test						
Triglyceride (mg/dL)	150.4 ± 63.2	139.3 ± 71.0	0.20	157.7 ± 95.5	167.2 ± 87.8	0.66
T- cholesterol (mg/dL)	197.2 ± 31.0	192.8 ± 26.5	0.21	202.0 ± 18.4	200.8 ± 19.5	0.74
HDL- cholesterol (mg/dL)	49.4 ± 11.3	53.5 ± 11.9	0.01	53.1 ± 11.0	52.5 ± 12.8	0.64
AI	3.1 ± 0.8	2.8 ± 1.0	0.003	3.0 ± 1.0	3.0 ± 0.9	0.72
Glucose (mg/dL)	96.6 ± 16.7	102.3 ± 14.4	0.13	96.8 ± 9.8	96.2 ± 8.1	0.77
Insulin (μU/mL)	10.8 ± 7.6	10.9 ± 13.7	0.97	17.6 ± 24.4	10.7 ± 9.8	0.19
HOMA-IR	2.7 ± 2.7	3.0 ± 4.6	0.81	4.3 ± 6.0	2.7 ± 2.8	0.22
HBA1C (%)	5.0 ± 0.5	4.9 ± 0.5	0.03	5.0 ± 0.4	4.9 ± 0.3	<0.001
24-h urinary sampling						
NaCl (g/day)	14.1 ± 3.7	11.2 ± 3.8	<0.01	14.1 ± 5.3	11.0 ± 4.8	0.03
K (g/day)	2.0 ± 0.5	1.95 ± 0.7	0.74	1.9 ± 0.4	1.9 ± 0.5	0.96
Na/K	4.8 ± 1.2	4.0 ± 1.4	0.01	4.9 ± 1.9	4.0 ± 2.1	0.11
Mg (mg/day)	101.0 ± 34.6	90.0 ± 53.8	0.22	95.5 ± 32.0	81.1 ± 25.6	0.01
Protein (g)	73.5 ± 15.0	70.7 ± 16.7	0.34	74.2 ± 15.8	67.6 ± 14.9	0.04
Isoflavoneds (µmol/day)	25.2 ± 19.1	53.0 ± 35.0	<0.001	19.1 ± 13.9	21.0 ± 23.9	0.70
Taurine (µmol/day)	1279.9 ± 595	1254.6 ± 547	0.79	1567.4 ± 1141	1773.1 ± 228	0.71

Data are means ± SD. Abbreviations: BMI—Body Mass Index, SBP—Systolic Blood Pressure, DBP—Diastolic Blood Pressure, AI—Atherogenic Index, K—potassium, Mg—magnesium.
